# Supplementary material for: Multi-omics delineation of cytokine-induced endothelial inflammatory states
Source: Commun Biol. 2023 May 15;6:525. doi: 10.1038/s42003-023-04897-w (PMC10184633; doi:10.1038/s42003-023-04897-w)
Supplement: Supplementary file 2 — Supplementary Information [file 42003_2023_4897_MOESM2_ESM.pdf]

Supplementary Figure 1

a

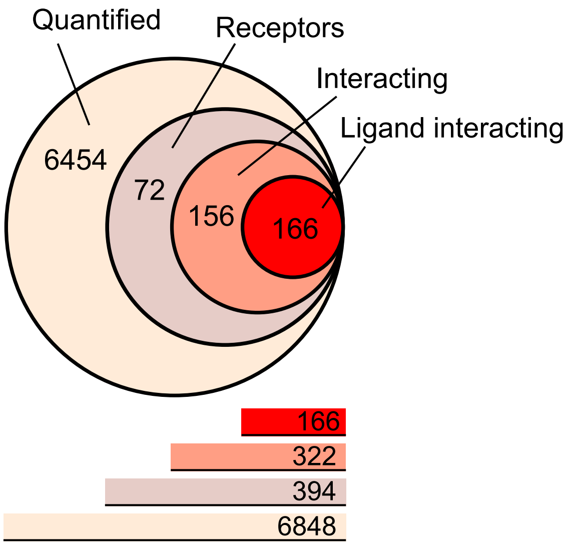

b

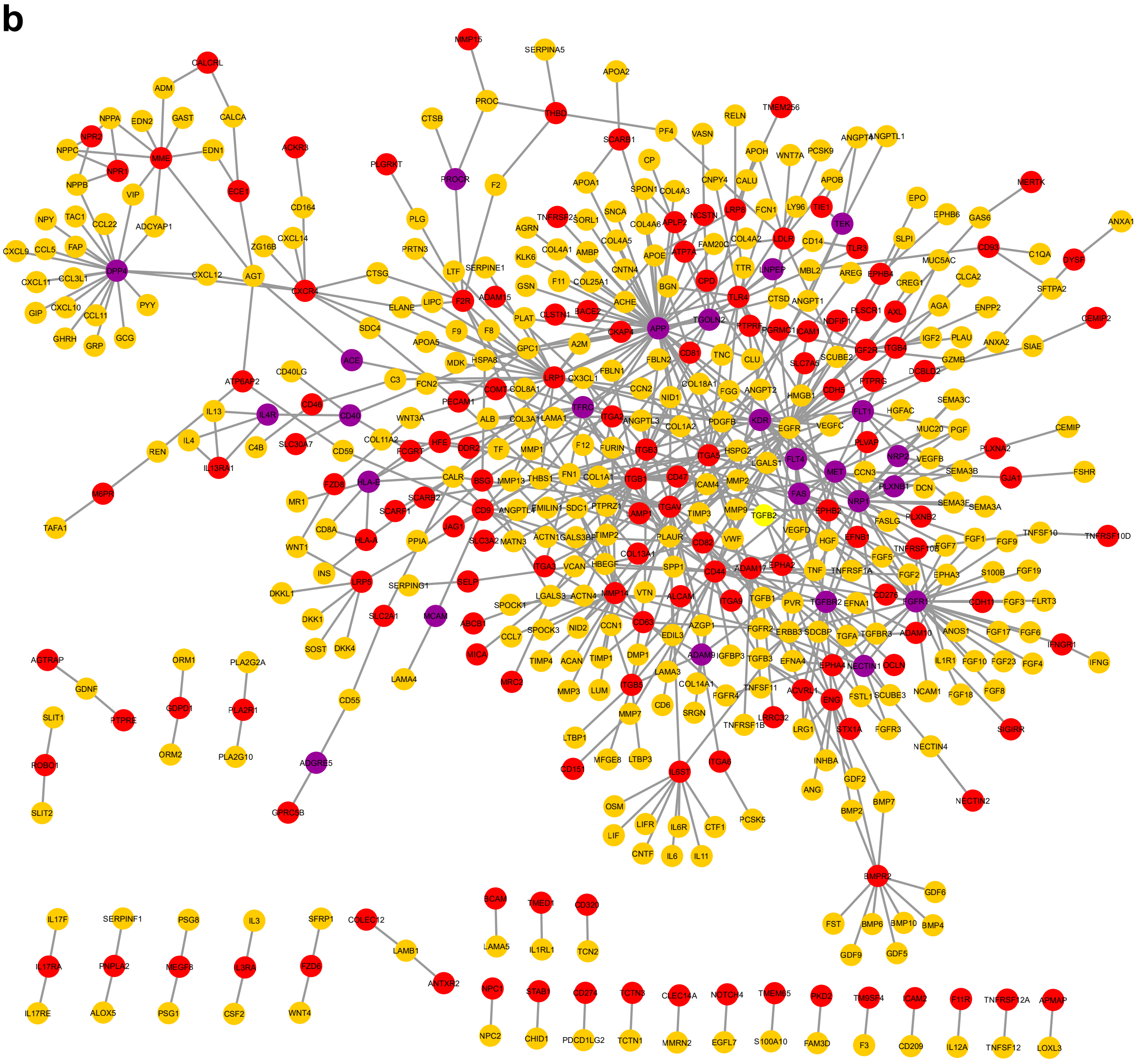

Supplementary Figure 1: In depth proteome of blood outgrowth endothelial cells

a Number of quantified proteins (Quantified), quantified proteins with 'extracellular' or 'GPI-anchor' topology domain Uniprot annotations (Receptors), receptors with STRING-DB interaction scores >0.4 (Interacting) and receptors interacting with Uniprot 'secreted' and 'signal' keywords or GO:CC 'extracellular space' and 'extracellular region' annotated targets (Ligand interacting) are indicated. Bars represent cumulative number of quantifications.

b Cytoscape interaction network of receptors and potential ligands (Red dots: receptors, yellow dots: ligands, purple dots: proteins fulfilling both receptor & ligand criteria), edges represent STRING-DB scores, nodes are labeled with protein names.

Supplementary Figure 2

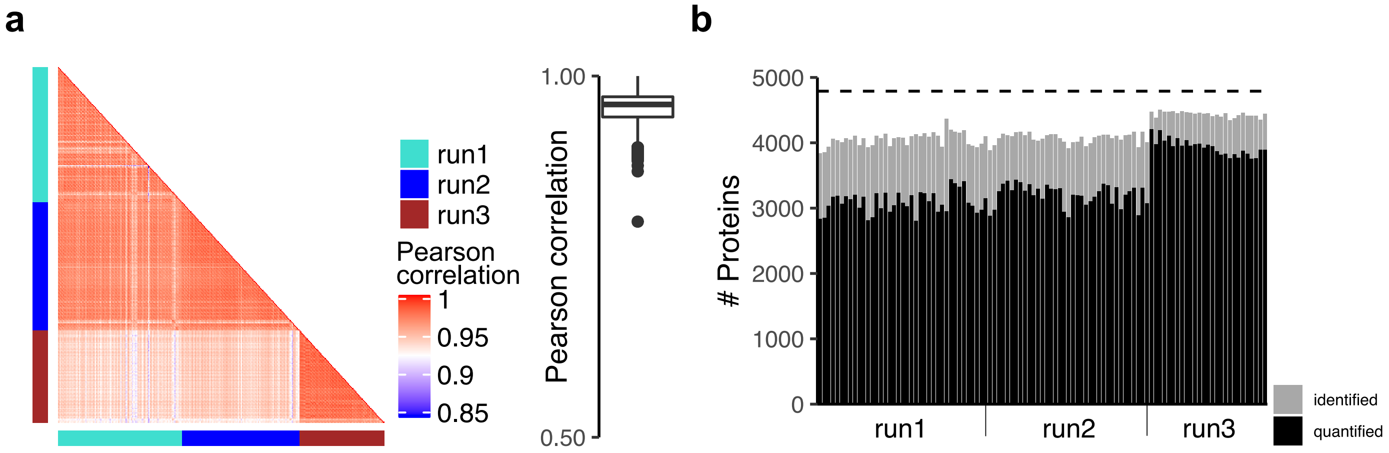

Supplementary Figure 2: Label free quantitative proteomics of cytokine library screening

a Pearson correlation coefficients of cytokine library screening. Color gradient scale represents Pearson correlation coefficients. Separate mass spectrometric runs are indicated (teal: run 1, blue: run 2, brown: run 3). Boxplot shows the overall distribution of Pearson correlation coefficients.

b Number of identified (grey bars) and quantified (black bars) proteins.

Supplementary Figure 3

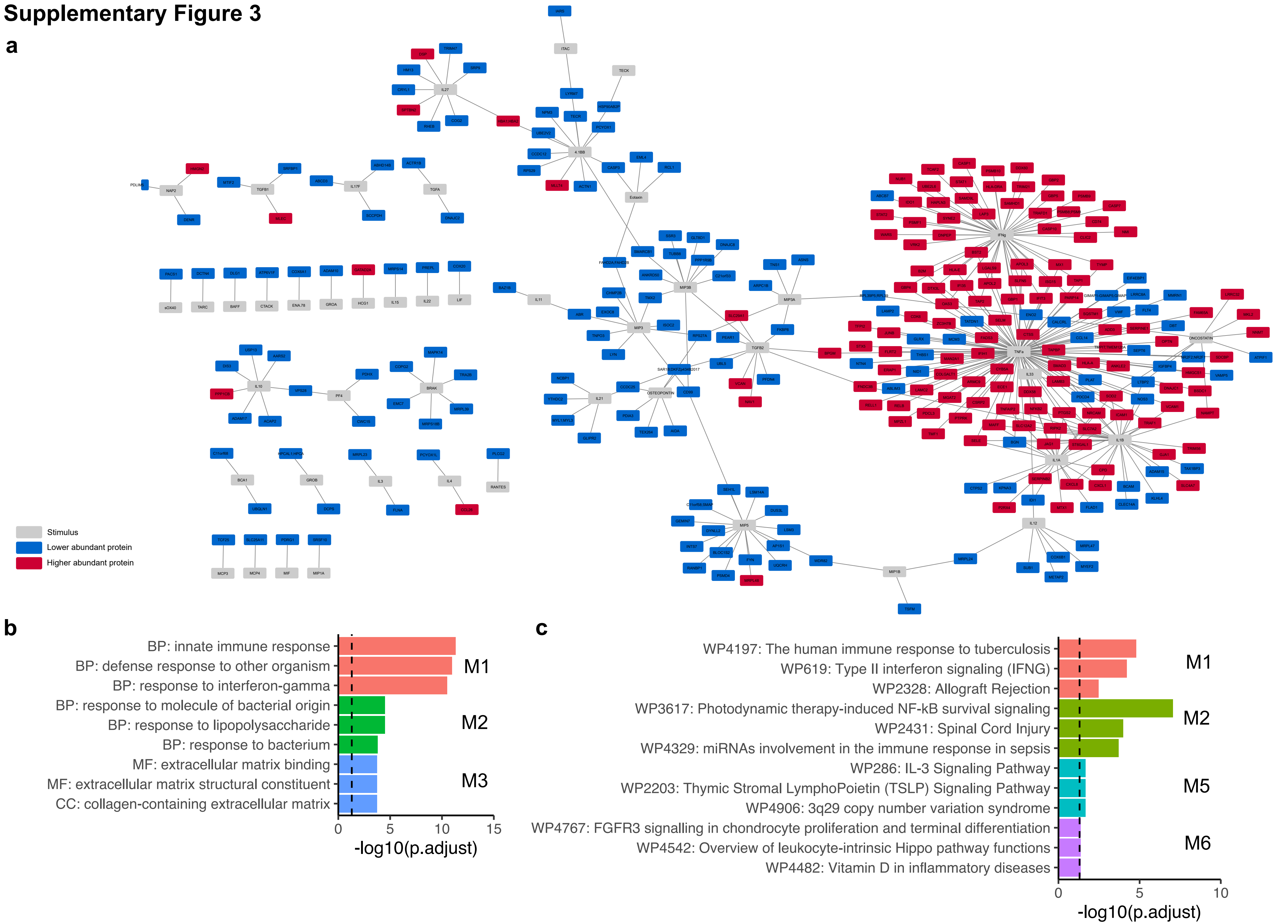

Supplementary Figure 3: Cytokine-EC proteomic response profiling

a Network visualizing connections between cytokine stimuli and statistically signi ficantly abundant proteins. Grey nodes: cytokine stimuli, blue nodes: proteins with reduced abundances, red nodes: proteins with increased abundances.

b Bar plot of top 3 enriched GeneOntology terms per module. Colors represent modules as indicated.

c Bar plot of top 3 enriched enriched pathways per module based on WikiPathway annotations. Colors represent modules as indicated.

Supplementary Figure 4

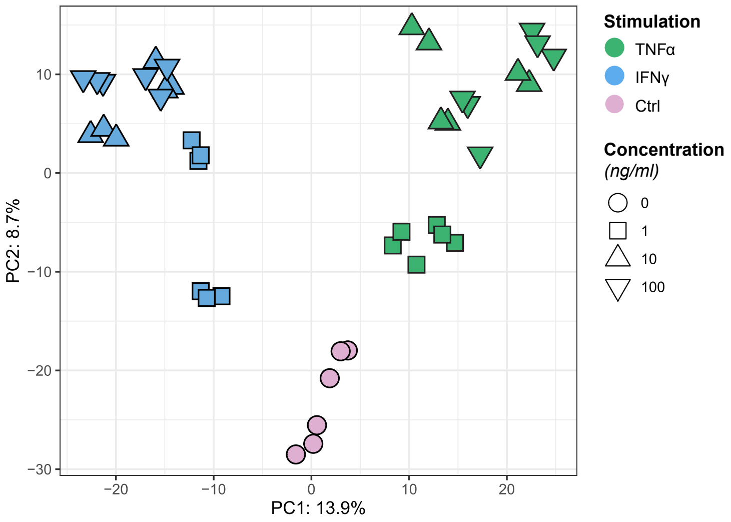

Supplementary Figure 4: Proteomic profiles of concentration ranges cytokine stimuli

PCA plot of proteomes of unstimulated BOECs (pink circles) or BOECs stimulated with increasing concentrations TNFα (green) and IFNγ (blue). Shape indicates concentration 1 ng/ml (square), 10 ng/ml (triangle), 100 ng/ml (upside down triangle).

Supplementary Figure 5

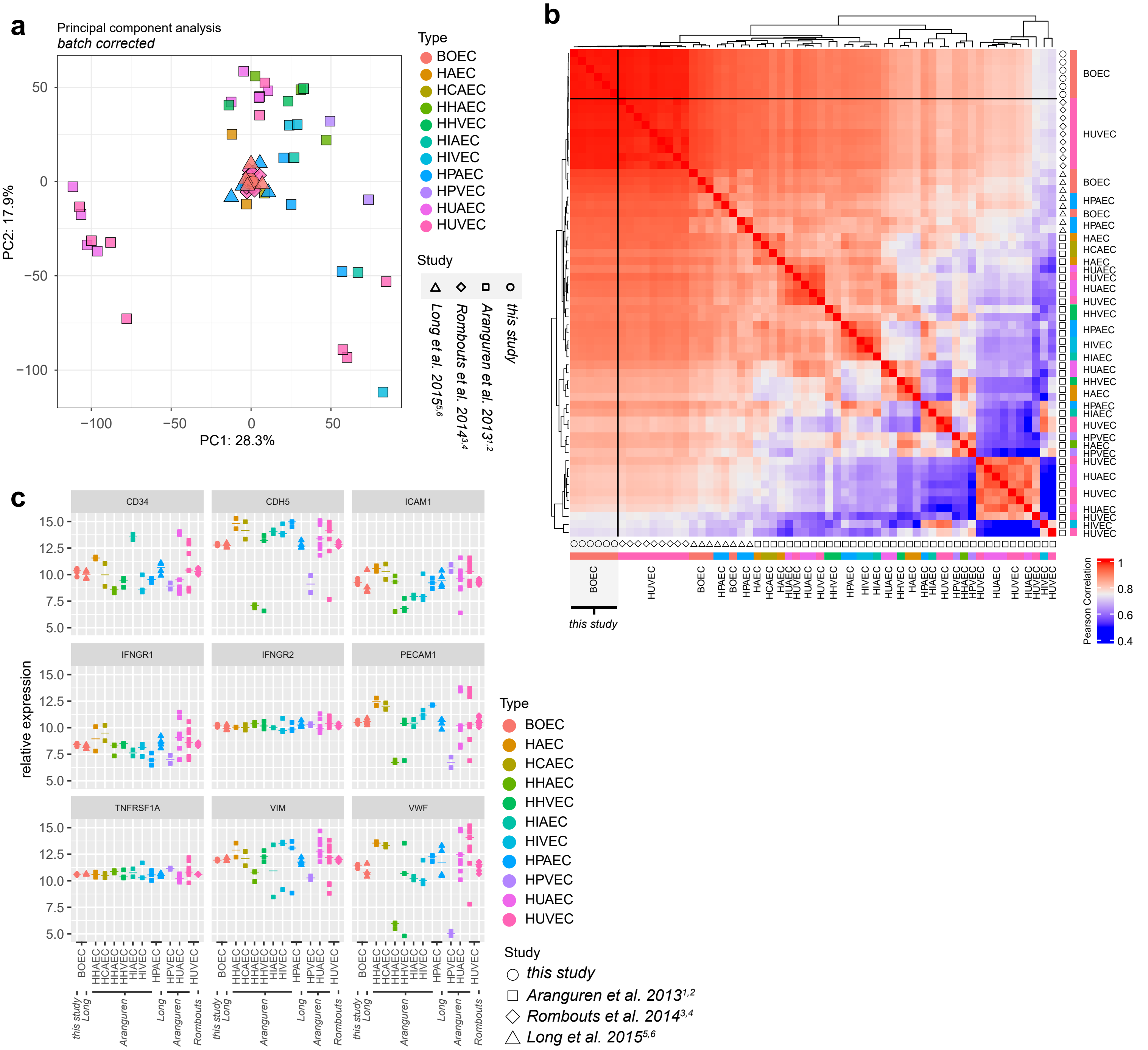

Supplementary Figure 5: Transcriptome comparison of steady state EC types

a PCA plots of EC types from different studies after batch correction. BOEC (blood outgrowth) (n=6), HAEC (aortic) (n=4), HCAEC (cardiac artery) (n=2), HHVEC (hepatic vein) (n=2), HIAEC (iliac artery) (n=2), HIVEC (iliac vein) (n=3), HPAEC (pulmonary artery) (n=6), HPVEC (pulmonary vein) (n=2), HUAEC (umbilical artery) (n=8), HUVEC (umbilical vein) (n=17) and HHAEC (hepatic artery) (n=2). EC types are indicated by color, shape indicates study data was derived from, this study (circle), Aranguren et al. 2013 (square), Rombouts et al. 2014 (diamonds), Long et al. 2015 (triangle)

b Heatmap showing pearson correlation between samples. Color gradient scale represents pearson correlation. Samples in this study are highlighted by black lines.

c Relative RNA expression levels of key EC markers between EC types. Points indicate relative expression of each sample, bar indicates average of EC types within a study.

Supplementary Figure 6

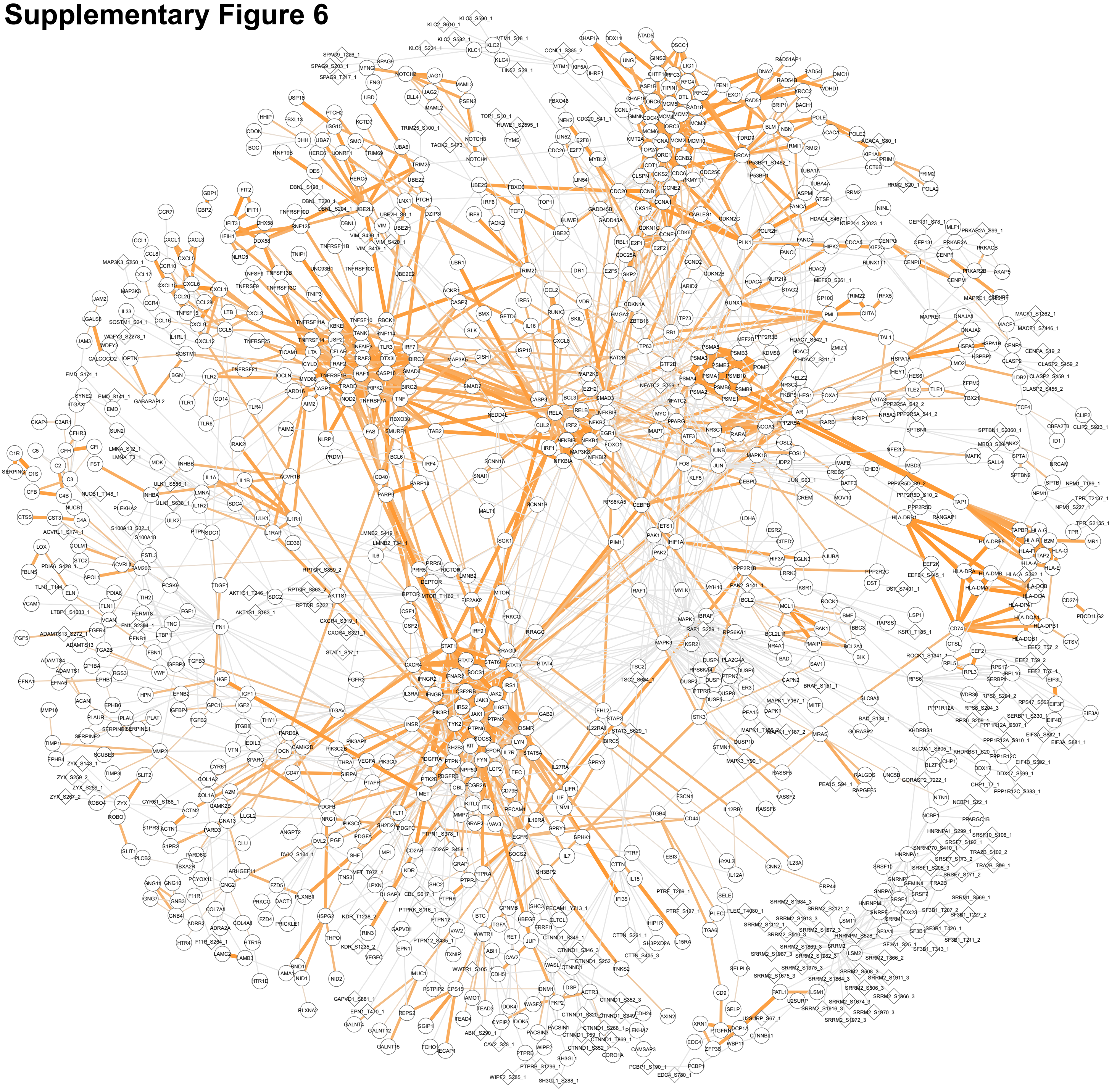

Supplementary Figure 6: Inflammation network

Interaction network of all regulated mRNAs/proteins and phosphosites, filtered by high confidence interactions, nodes represent transcripts/proteins and phosphosites and are labeled with gene names and phosphosites.

Supplementary Figure 7

a

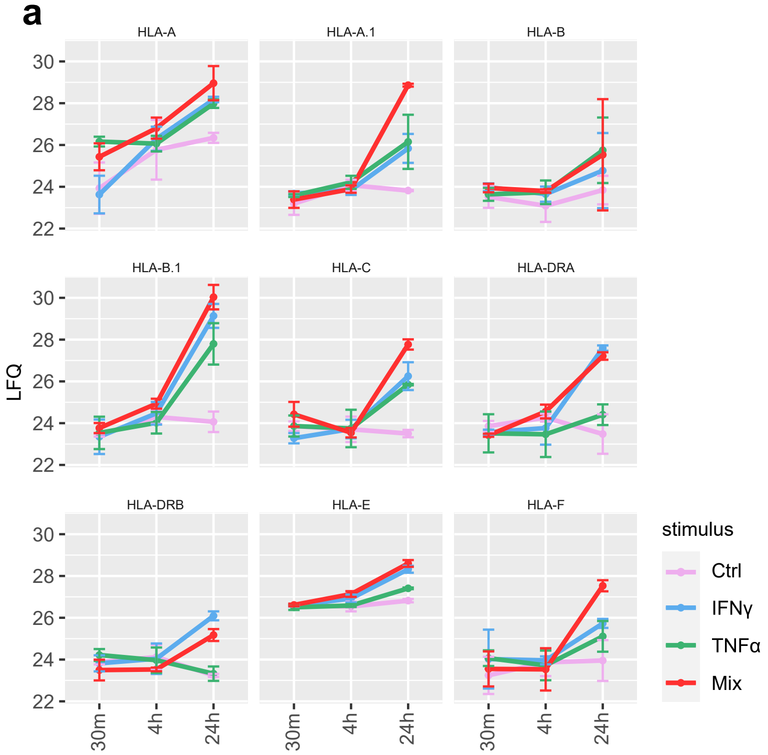

b

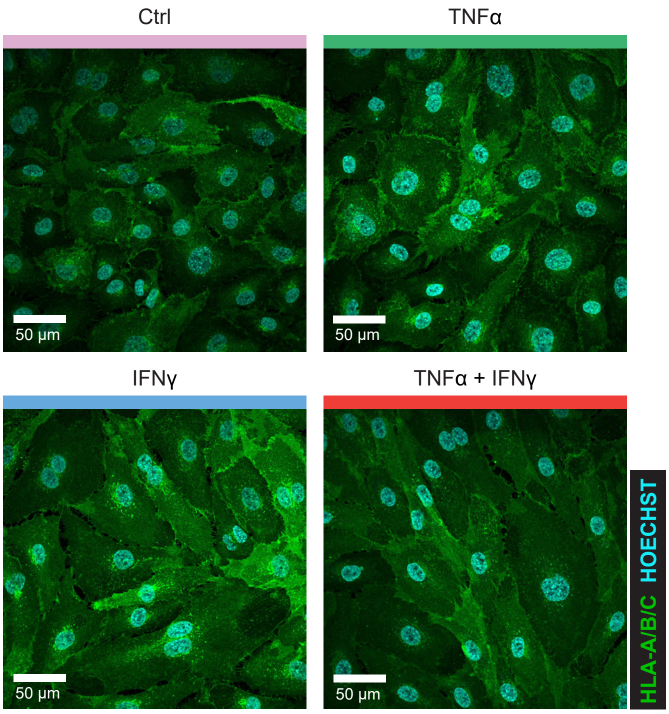

Supplementary Figure 7: HLA expression endothelial cells

a Detected HLAs in LFQ proteomic workflow. Line plots of HLA LFQ levels after TNFα (green), IFNγ (blue) and TNFα + IFNγ (red) stimulation or unstimulated (pink). Circles indicate median, error bars show standard deviation (n = 3 biological replicates).

b MHCI distribution across EC cell membrane. Confocal images of HLA-A/B/C immunostaining in unstimulated ECs (Ctrl, pink) and stimulated with TNFα (green), IFNγ (blue), or TNFα + IFNγ (red). HLA-A/B/C staining is depicted in green, Hoechst staining in cyan. Representative experiment shown (n = 3 biologically independent experiments). Upper limit of the display range were adjusted equally across images for visualization purposes.

Supplementary Figure 8

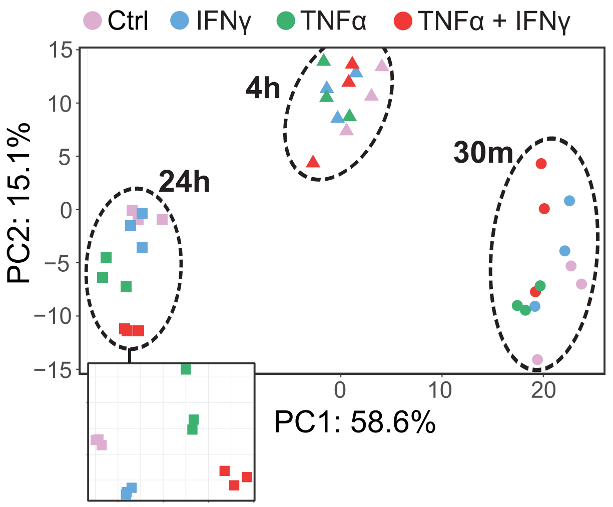

Supplementary Figure 8: Variation in secreted proteins is induced by time and stimulus PCA plot of secretomes of unstimulated BOECs (pink) or BOECs stimulated with TNFα (green), IFNγ (blue) or TNFα + IFNγ (red). Time of stimulation is indicated by shapes, 30 m (circle), 4h (triangle), 24h (square). Seperate PCA of samples at 24h is shown in detail

Supplementary table 1

Proteins	Peprotech Catalog	HNCG SYMBOL	Proteins	Peprotech Catalog	HNCG SYMBOL
4-1BB	310-11	TNFSF9	IL-4	200-04	IL4
BAFF	310-13	TNFSF13B	IL-5	200-05	IL5
BCA-1	300-47	CXCL13	IL-6	200-06	IL6
BRAK	300-50	CXCL14	IL-7	200-07	IL7
CTACK	300-54	CCL27	IL-8	200-08M	CXCL8
CXCL16	300-55	CXCL16	IL-9	200-09	IL9
EGF	AF-100-15	EGF	IP-10	300-12	CXCL10
ENA-78	300-22	CXCL5	I-TAC	300-46	CXCL11
Eotaxin	300-21	CCL11	LD78β	300-56	CCL3L1
Eotaxin-2	300-33	CCL24	LEC	300-44	CCL16
Eotaxin-3	300-48	CCL26	LIF	300-05	LIF
Exodus-2	300-35	CCL21	LIGHT	310-09B	TNFSF14
FGF-acidic	100-17A	FGF1	Lymphotactin	300-20	XCL1
FGF-basic	100-18B	FGF2	MCP-1	300-04	CCL2
Fractalkine	300-31	CX3CL1	MCP-2	300-15	CCL8
GCP-2	300-41	CXCL6	MCP-3	300-17	CCL7
G-CSF	300-23	CSF3	MCP-4	300-24	CCL13
GM-CSF	300-03	CSF2	M-CSF	300-25	CSF1
GRO-α/MGSA	300-11	CXCL1	MDC	300-36	CCL22
GRO-β	300-39	CXCL2	MIF	300-69	MIF
GRO-γ	300-40	CXCL3	MIG	300-26	CXCL9
HCC-1	300-38	CCL14	MIP-1α	300-08	CCL3
HGF	100-39H	HGF	MIP-1β	300-09	CCL4
I-309	300-37	CCL1	MIP-3	300-29	CCL23
IFN-γ	300-02	IFNG	MIP-3α	300-29A	CCL20
IL-10	200-10	IL10	MIP-3β	300-29B	CCL19
IL-11	200-11	IL11	MIP-5	300-43	CCL15
IL-12	200-12H	IL12A	NAP-2	300-14	PPBP
IL-13	200-13	IL13	Oncostatin	300-10	OSM
IL-15	200-15	IL15	Osteopontin	120-35	SPP1
IL-16	200-16	IL16	PF-4	300-16	PF4
IL-17A	200-17	IL17A	RANTES	300-06	CCL5
IL-17B	200-28	IL17B	Resistin	450-19	RETN
IL-17F	200-25	IL17F	sCD27	310-30	CD70
IL-1RA	200-01RA	IL1RN	sCD30	450-42	TNFSF8
IL-1α	200-01A	IL1A	sCD40	310-02	CD40LG
IL-1β	200-01B	IL1B	SDF-1β	300-28B	CXCL12
IL-2	200-02	IL2	sOX40	310-28	TNFSF4
IL-21	200-21	IL21	sRANK	310-01	TNFSF11
IL-22	200-22	IL22	TARC	300-30	CCL17
IL-23	200-23	IL23A	TECK	300-45	CCL25
IL-27	200-38	IL27	TGF-α	100-16A	TGFA
IL-3	200-03	IL3	TGF-β1	100-21	TGFB1
IL-33	200-33	IL33	TGF-β2	100-35B	TGFB2
IL-34	200-34	IL34	TGF-β3	100-36E	TGFB3
IL-36γ	200-36G	IL36G	TNF-α	300-01A	TNF

Supplementary table 1

Overview of the cytokine library used in this study

Supplementary table 2

Cytokine library screen

	Sex	Age
MIX I	F	40
	M	58
	F	59[a]
MIX II	M	50
	F	na[b]
	F	33
MIX III	na	na
	M	57
	na	na

Cytokine library reruns

	Sex	Age
MIX I	F	59[a]
	M	49[d]
	F	40
MIX II	M	58
	F	26[c]
	M	41
MIX III	M	60
	F	44
	F	na[b]

Multi-omics experiment

	Sex	Age
MIX	F	59[a]
	F	26[c]
	M	49[d]

Secretome experiment

	Sex	Age
MIX	M	63
	F	48
	M	48

Supplementary table 2

Overview of age and sex of donors from which BOECs were isolated that were used in main experiments. x indicates the same donor. na indicates donor information was not available. In the cytokine library screen several conditions rerun. All cell culture plates used in both original and rerun samples, contained TNFα and IFNγ stimulated conditions.

Supplementary References

1. Aranguren, X. L. et al. Unraveling a novel transcription factor code determining the human arterial-specific endothelial cell signature. Blood 122, 3982–92 (2013).
2. Xabier Agirre, Xabier Aranguren, Victor Segura, Alberto Pascual-Montano, Felipe Prósper & Aernout Luttun (2014). UNRAVELING A NOVEL TRANSCRIPTION FACTOR CODE INDUCTIVE FOR THE HUMAN ARTERIAL-SPECIFIC ENDOTHELIAL CELL SIGNATURE. BioStudies, E-GEOD-43475. Retrieved from https://www.ebi.ac.uk/biostudies/arrayexpress/studies/E-GEOD-43475
3. Rombouts, C. et al. Transcriptomic profiling suggests a role for IGFBP5 in premature senescence of endothelial cells after chronic low dose rate irradiation. Int. J. Radiat. Biol. 90, 560–74 (2014).
4. Roel Quintens & An Aerts (2018). Microarray analysis of human umbilical vein endothelial cells (HUVECs) chronically irradiated with low dose-rate of ionizing radiation for one, three and six weeks. BioStudies, E-MTAB-6521. Retrieved from https://www.ebi.ac.uk/biostudies/arrayexpress/studies/E-MTAB-6521
5. Long, L. et al. Selective enhancement of endothelial BMPR-II with BMP9 reverses pulmonary arterial hypertension. Nat. Med. 21, 777–85 (2015).
6. Stefan Gräf & Nicholas Morrell (2015). BMP9_BMPR2_PAEC_BOEC. BioStudies, E-MTAB-2495. Retrieved from https://www.ebi.ac.uk/biostudies/arrayexpress/studies/E-MTAB-2495
