# Supplementary material for: Multi-omics delineation of cytokine-induced endothelial inflammatory states
Source: Commun Biol. 2023 May 15;6:525. doi: 10.1038/s42003-023-04897-w (PMC10184633; doi:10.1038/s42003-023-04897-w)
Supplement: Supplementary file 3 — Description of Additional Supplementary Files [file 42003_2023_4897_MOESM3_ESM.pdf]

Description of Additional Supplementary Files

File name: Supplementary Data 1

Description: Source proteomics data and analysis behind figure 1

File name: Supplementary Data 2

Description: Source proteomics data and analysis behind figure 2

File name: Supplementary Data 3

Description: Source proteomics data behind figure 3

File name: Supplementary Data 4

Description: Source (phospho-)proteomics & mRNA expression data and analysis behind figure 4-7

File name: Supplementary Data 5

Description: Source proteomics data and analysis behind figure 8
