# Supplementary material for: Multi-omics delineation of cytokine-induced endothelial inflammatory states
Source: Commun Biol. 2023 May 15;6:525. doi: 10.1038/s42003-023-04897-w (PMC10184633; doi:10.1038/s42003-023-04897-w)
Supplement: Supplementary file 9 — Reporting Summary [file 42003_2023_4897_MOESM9_ESM.pdf]

nature portfolio

Corresponding author(s): Maartje van den Biggelaar, PhD

Last updated by author(s): Apr 19, 2023

Reporting Summary

Nature Portfolio wishes to improve the reproducibility of the work that we publish. This form provides structure for consistency and transparency in reporting. For further information on Nature Portfolio policies, see our Editorial Policies and the Editorial Policy Checklist.

Statistics

For all statistical analyses, confirm that the following items are present in the figure legend, table legend, main text, or Methods section.

n/a	Confirmed	
☐	☒	The exact sample size (*n*) for each experimental group/condition, given as a discrete number and unit of measurement
☐	☒	A statement on whether measurements were taken from distinct samples or whether the same sample was measured repeatedly
☐	☒	The statistical test(s) used AND whether they are one- or two-sided<br>*Only common tests should be described solely by name; describe more complex techniques in the Methods section.*
☒	☐	A description of all covariates tested
☐	☒	A description of any assumptions or corrections, such as tests of normality and adjustment for multiple comparisons
☐	☒	A full description of the statistical parameters including central tendency (e.g. means) or other basic estimates (e.g. regression coefficient) AND variation (e.g. standard deviation) or associated estimates of uncertainty (e.g. confidence intervals)
☐	☒	For null hypothesis testing, the test statistic (e.g. *F*, *t*, *r*) with confidence intervals, effect sizes, degrees of freedom and *P* value noted<br>*Give P values as exact values whenever suitable.*
☒	☐	For Bayesian analysis, information on the choice of priors and Markov chain Monte Carlo settings
☒	☐	For hierarchical and complex designs, identification of the appropriate level for tests and full reporting of outcomes
☒	☐	Estimates of effect sizes (e.g. Cohen's *d*, Pearson's *r*), indicating how they were calculated

Our web collection on statistics for biologists contains articles on many of the points above.

Software and code

Policy information about availability of computer code

Data collection	MS data collection was performed on a nanoscale C18 reverse chromatography coupled on-line to either an Orbitrap Fusion Lumos Tribrid or Orbitrap Fusion Tribrid mass spectrometer (Thermo Fisher). RNA sequencing data collection was performed by GeneWiz (Azenta life sciences)
Data analysis	MS data was processed with Maxquant (1.6.2.10) using the Andromeda engine while RNA sequencing was processed with STAR (2.7.8a). Data was analysed in R (3.5.2) / Rstudio (1.1.456), for specific packages used see methods. Data was visualized in Cytoscape (3.8.0)

For manuscripts utilizing custom algorithms or software that are central to the research but not yet described in published literature, software must be made available to editors and reviewers. We strongly encourage code deposition in a community repository (e.g. GitHub). See the Nature Portfolio guidelines for submitting code & software for further information.

Data

Policy information about availability of data

All manuscripts must include a data availability statement. This statement should provide the following information, where applicable:

- Accession codes, unique identifiers, or web links for publicly available datasets
- A description of any restrictions on data availability
- For clinical datasets or third party data, please ensure that the statement adheres to our policy

The MS raw and output files have been deposited to the ProteomeXchange Consortium via the PRIDE partner repository with the dataset identifier PXD036582. mRNA sequencing data has been deposited in NCBI's Gene Expression Omnibus and is accessible through GE Series accession number (GSE213111).

Human research participants

Policy information about studies involving human research participants and Sex and Gender in Research.

Reporting on sex and gender	No human research participants were involved in this study
Population characteristics	-
Recruitment	-
Ethics oversight	-

Note that full information on the approval of the study protocol must also be provided in the manuscript.

Field-specific reporting

Please select the one below that is the best fit for your research. If you are not sure, read the appropriate sections before making your selection.

☒ Life sciences ☐ Behavioural & social sciences ☐ Ecological, evolutionary & environmental sciences

For a reference copy of the document with all sections, see nature.com/documents/nr-reporting-summary-flat.pdf

Life sciences study design

All studies must disclose on these points even when the disclosure is negative.

Sample size	Cells from 19 different donors were used, pooled per three for experiments. Stimulations were performed in 3 biological replicates
Data exclusions	No data was excluded
Replication	Proteomic signatures of unstimulated, TNFa and IFNy stimulated cells were reproducible throughout experiments and endothelial cell pools.
Randomization	Endothelial cell pools were made randomly
Blinding	No blinding was applied

Reporting for specific materials, systems and methods

We require information from authors about some types of materials, experimental systems and methods used in many studies. Here, indicate whether each material, system or method listed is relevant to your study. If you are not sure if a list item applies to your research, read the appropriate section before selecting a response.

Materials & experimental systems

n/a	Involved in the study	
☐	☒	Antibodies
☐	☒	Eukaryotic cell lines
☒	☐	Palaeontology and archaeology
☒	☐	Animals and other organisms
☒	☐	Clinical data
☒	☐	Dual use research of concern

Methods

n/a	Involved in the study	
☒	☐	ChIP-seq
☒	☐	Flow cytometry
☒	☐	MRI-based neuroimaging

Antibodies

Antibodies used	anti HLA-DR, L243 (InVivoMAb, BE0306, 688119S1), anti pan-HLA-A,-B,-C, W6/32 (ATCC, HB-95), secondary Alexa Fluor 488 conjugated antibody (Invitrogen, A21200, 1696214)
Validation	HLA-DR (L243)<br>RRID: AB_2736986, the antibody registry<br>pan HLA-A,-B,-C (W6/32)<br>-Brodsky FM, Parham P. Monomorphic anti-HLA-A,B,C monoclonal antibodies detecting molecular subunits and combinatorial determinants. J. Immunol. 128: 129-135, 1982. PubMed: 6172474

Barnstable CJ, et al. Production of monoclonal antibodies to group A erythrocytes, HLA and other human cell surface antigens -- new tools for genetic analysis. Cell 14: 9-20, 1978. PubMed: 667938

Eukaryotic cell lines

Policy information about cell lines and Sex and Gender in Research

Cell line source(s)	Blood derived endothelial cells were isolated from healthy donors (Martin-Ramirez, J. et al., Establishment of outgrowth endothelial cells from peripheral blood. Nat. Protoc. 7, 1709–15 (2012)). For overview of cell source donor see supplemental table 2
Authentication	Endothelial cell marker VWF expression was checked by immunostaining for all isolated cells
Mycoplasma contamination	Cells were tested negative for mycoplasm after isolation
Commonly misidentified lines (See ICLAC register)	This study does not use commonly misidentified cell lines
